# Barrier Effects of a poly (lactic acid/caprolactone) Bilayer Membrane for Guided Bone Regeneration on Skull without Periosteum of Adult-Senior Staged Rats

**DOI:** 10.4317/jced.62490

**Published:** 2025-02-01

**Authors:** Masaru Murata, Md. Arafat Kabir, Naohiro Horie, Kenji Yokozeki, Mamata Shakya, Kaoru Kusano

**Affiliations:** 1 Division of Oral Regenerative Medicine, School of Dentistry, Health Science University of Hokkaido, Japan; 2 Division of Reconstructive Surgery for Oral and Maxillofacial Region, School of Dentistry, Health Sciences University of Hokkaido, Japan; 3 Department of Oral Implantology, School of Dentistry, Osaka Dental University, Japan

## Abstract

**Background:**

Vertical and horizontal bone augmentation is one of the most challenging techniques in bone engineering. The use of barrier membranes and scaffolds in guided bone regeneration (GBR) procedures is a common approach for the treatment of lost bone around teeth and dental implants. The aim of this study was to estimate the barrier effects of a synthetic poly (lactic acid/caprolactone) [P(LA/CL)] bilayer membrane for GBR, compared to a porcine collagen bilayer membrane, in the vertical augmentation model on 10–12-months old rat skull without periosteum.

**Material and Methods:**

The hydroxyapatite (HAp) block (diameter: 4 mm, height: 3 mm, porosity:75%, average pore size:150 μm) was placed on the rat skull without a periosteum. The P(LA/CL) membrane (solid layer: 25 μm, porous layer: 175 μm) or the collagen membrane (solid layer, porous layer) was applied onto the HAp block. At 3, 6, and 12 weeks after the surgery, the incised tissues were fixed, decalcified, and stained with hematoxylin and eosin for histological evaluation.

**Results:**

The P(LA/CL) membrane remained until 12 weeks and could achieve barrier effects to inhibit cellular invasion from the repositioned soft tissues. Local bone formation occurred in the interconnected pores of HAp at 6 weeks. On the other hand, the collagen membrane did not inhibit cellular invasion for its expansion until 3 weeks, and was absorbed until 6 weeks. Histomorphometrically, bone in the P(LA/CL)/HAp at 6 and 12 weeks occupied 8.3 % and 10.0 %, respectively, while bone was not formed in the pores of the upper half area in the collagen/HAp.

**Conclusions:**

The results in the biomimetic model indicated that the P(LA/CL) membrane might be effective in GBR as an occlusive and absorbable membrane.

** Key words:**Guided bone regeneration (GBR), bone, augmentation, absorbable, membrane, collagen, hy-droxyapatite; P(LA/CL), biomimetic.

## Introduction

Membranes are a key factor to the GBR procedure, being able to function as a barrier against fibroblasts for osteogenic environments. The cell-barrier membranes are divided into absorbable and non-absorbable (1-4). The non-absorbable membranes require a second surgical procedure for the removal. Therefore, absorbable membranes are strongly needed in the field of local bone regeneration for one-staged surgery and avoiding additional morbidity such as a second surgery increasing patient’s pain and costs. Among the absorbable membranes, collagen-based materials have been used widely (2-4). Although animal-derived collagen materials are biocompatible medical devices, it is difficult to control the elasticity and the absorption rate of the collagen-based materials. The enzymatic activity such as matrix metalloproteinases (MMPs) causes the collagen membrane to digest rapidly and decreases the cell-barrier function. Therefore, a novel polymer membrane composed of poly (lactic acid) (PLA) and poly (caprolactone) (PCL) has been developed to act as a cell-barrier membrane during bone formation for GBR in Japan (5), and the bio-absorbable GBR membrane is a poly (lactic acid /caprolactone) (P(LA/CL)) bilayer membrane without animal products (5,6). The degradation rate of the P(LA/CL) bilayer membrane was 40% and 55% in weight at 12 and 26 weeks, respectively (5). Already, the safety and feasibility of the P(LA/CL) membrane with carbonate apatite granules and autogenous bone was clinically assessed for GBR in bone defects surrounding dental implants in human pilot studies (7,8). However, the fate and behavior of the P(LA/CL) membrane in the applied sites were not mentioned in the clinical papers.

GBR is the popular technique for horizontal and/or vertical bone augmentation in implant dentistry (9). The vertical augmentation is especially one of the most challenging techniques in bone engineering. Although there have been many animal studies related with GBR, almost all studies have used the GTR membranes in bone defect (inlay) models with periosteum in young-adult rats (10,11). Moreover, almost patients related with bone loss by periodontal disease or implant rehabilitation are in the adult-senior stage, not young. In the present study, therefore, aged rats in the adult-senior stage were used, and we focused on the barrier effects of the P(LA/CL) membrane against non-osteogenic soft tissues without periosteum as clinically biomimetic model.

The aim of this study was to estimate the barrier effects of a novel P(LA/CL) bilayer membrane for GBR, compared to a porcine collagen bilayer membrane, histologically in the vertical (onlay) model on skull without a periosteum of 10-12 months old rats.

## Material and Methods

1. Biomaterials 

**Figure 1:**
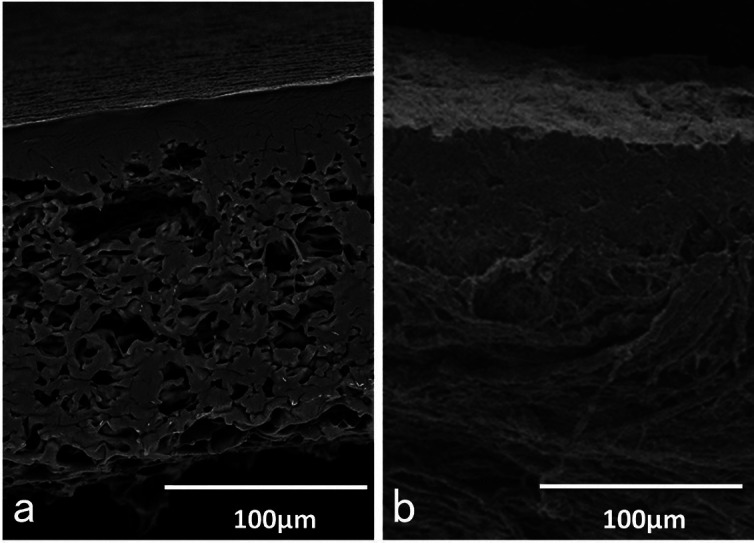
Images of scanning electron microscopy of absorbable bilayer membranes. (a) P(LA/CL) bilayer membrane. Upper layer; solid structure (25 μm). Lower layer; multi-porous (175 μm). Note: Upper solid layer contact with repositioned soft tissues. (b) Collagen bilayer membrane.

Absorbable bilayer membranes and non-absorbable synthetic hydroxyapatite (HAp) block were used in this study. The HAp block (diameter: 4 mm, height: 3 mm, porosity: 75%, average pore size: 150 μm) (NEOBONE®, CoorsTek GK, Japan) was placed on the rat skull without a periosteum. As an experimental group, a poly (lactic acid /caprolactone: P(LA/CL)) bilayer membrane (Cytrans®Elashield, GC Corp, Tokyo, Japan, Fig. 1 (a)) was applied onto the HAp block for GBR. The synthetic P(LA/CL) membrane consists of a bilayer structure (totally 200 μm in thickness) with solid (25 μm) and porous (175 μm) layers (3,4). As a control group, a collagen bilayer membrane (Bio-Guide®, Gaistrich Pharma, Wolhusen, Switzerland, Fig. 1 (b)) was used onto the HAp block. The non-cross-linked collagen membrane derived from porcine dermis exhibited a dense structure in the upper layer and a porous structure in the lower layer (totally 440 μm in thickness) (12,13). Both membranes were cut into a round shape (diameter: 8 mm) by a sterilized biopsy punch (BP-80F, Kai medical, Japan) before use.

2. Animal Experiment 

Twenty-seven Wistar rats (10-12 months old, female, weight range: 280-320 g) were used to evaluate *in vivo* effects of each barrier membrane onto the HAp block. The animal experiment was examined by the Institutional Animal Care and Use Committee of the Health Sciences University of Hokkaido (approval number: 21-047).

3. Surgical Procedures

**Figure 2:**
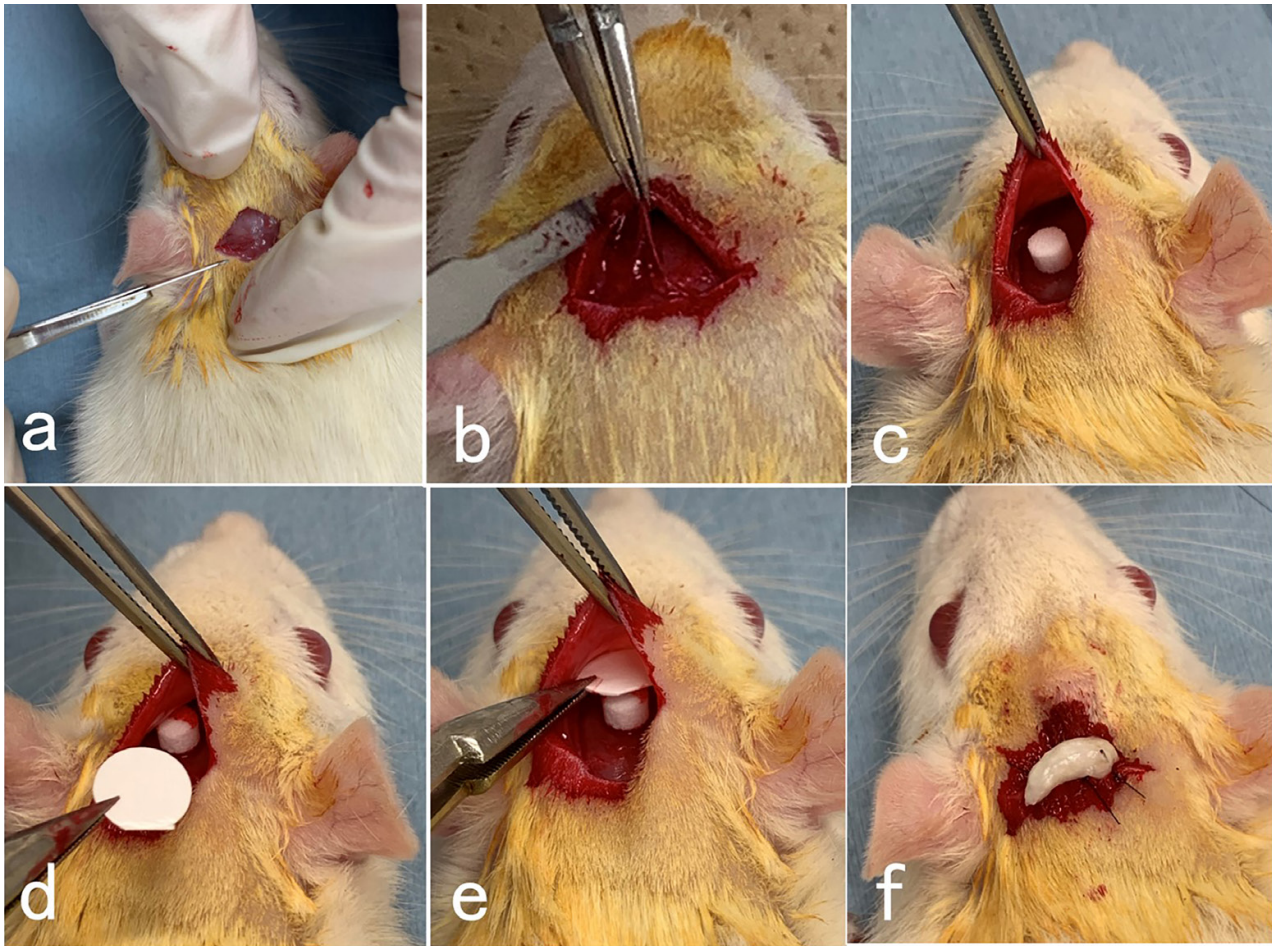
Photography of surgical procedures on adult-senior staged rat skull in GBR model. (a) Incision and exposure of periosteum. (b) Removal of periosteum from parietal bone after elevation of scalp. (c) Cylindrical HAp block (diameter: 4 mm, height: 3 mm) on exposed parietal bone without periosteum. (d) Barrier membrane insertion (diameter: 8 mm). (e) Membrane between HAp and soft tissues without periosteum. Note: solid (dense) layer of membrane contacted with soft tissues, (f) Antibiotics paste on skin sutured with 5/0 nylon.

The rats were divided randomly into two groups, as follows: the control group, the collagen membrane onto the HAp block on the exposed parietal bone without periosteum (n=15); the experimental group, the p(LA/CL) membrane onto the HAp block (n=12). Wistar rats were subjected to intraperitoneal anesthesia with pentobarbital sodium (4 mg/100 g body weight). Surgical operations were performed under the general anesthesia, the head hairs were shaved and an incision allowing reflection of a full-thickness flap was made in the scalp in the sagittal plane (Fig. 2 (a)). The head skin was elevated with galea, and the periosteum was removed from the outer cortical plate of parietal bone (Fig. 2 (b)). The HAp block was implanted on the exposed skull without a periosteum (Fig. 2 (c)). Each membrane was placed onto the HAp block (Fig. 2 (d,e)) without fixation of the membrane by tack pins. The skull skin was sutured with 5/0 nylon sutures. Chloromycetin ointment was used after the sutures (Fig. 2 (f)). All surgical procedures were performed by the same surgeon. All rats were fed with a standard diet during the experimental period.

4. Histological Examination

At 3, 6, and 12 weeks after the surgery, the rats were sacrificed (4 rats as the control group and 5 rats as the experiment group at each time point) by cervical dislocation under an anesthetic overdose (ketamine at a dose 2–3-fold higher than the anesthetic dosage). After the incision of head skin, a surgical drill attached to an electrical hand motor piece was used to harvest the skull bone with both the implanted materials and the head skin.

The incised tissues were immersed in 20% neutral phosphate-buffered formalin solution, additional immersion by microwave treatment, and then fixed in 20% neutral phosphate-buffered formalin solution for 1 week. The fixed tissues were decalcified in 10% formic acid for 4 weeks, and rinsed overnight with running water. All the samples were dehydrated using ethanol (50%–100%) and processed for paraffin embedding (Vacuum Rotary, VRX-23, Mitsubishi, Tokyo, Japan). Later, histological sections (5 µm thickness) were prepared by a microtome (Yamato Rom 380, Tokyo, Japan) and stained with hematoxylin and eosin (HE) (Wako, Osaka, Japan). The HE-stained sections were examined by using an optical microscopy (Nikon Eclipse 80i, Nikon, Tokyo, Japan) for histological evaluation.

5. Histomorphometric Measurement

The middle longitudinal sections were selected from 3 blocks for the measurement. The concerning area was decided as the upper half area of total HAp. Explanted tissues were divided into following 4 parts: HAp, new bone, immature tissues (IT), and fibrous connective tissues (CT). The area of each tissue was outlined by the investigator using Image J software (Image J 1.46r, the National Institute of Health, Bethesda, Maryland, USA). A statistical analysis was conducted to compare the areas of HAp, new bone, immature tissues, and connective tissues at 3, 6, and 12 weeks. All measurements were performed by 2 investigators.

All data were presented as the mean ± standard deviation (SD). The statistical significance of the change in response was assessed by a paired t-test. Differences were considered significant at *P*<0.05. Statistical analysis was performed using a Windows computer with SPSS software (SPSS ver. 19, IBM, USA).

## Results

1. Histological Findings in P(LA/CL) /HAp on Skull without Periosteum

**Figure 3:**
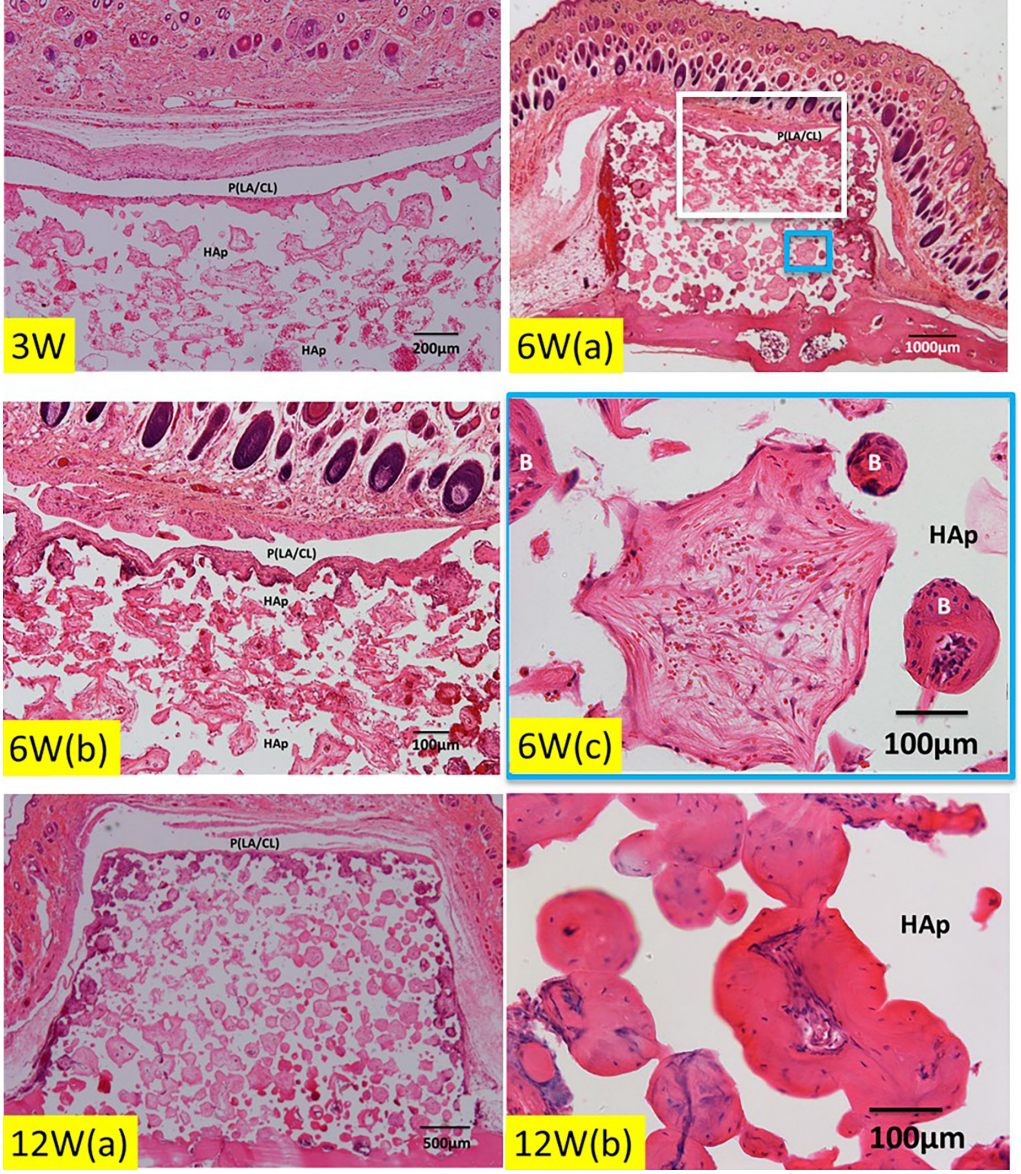
Histological images of P(LA/CL) /HAp on skull without periosteum. 3W; Fibrous connective tissues on P(LA/CL) membrane at 3 weeks. Red blood cells and body fluid in pores of HAp. Note: P(LA/CL) and HAp bulk showing clear spaces after completely demineralization in HE. 6W (a) Total view at 6 weeks. Hair and fibrous connective tissues on P(LA/CL). 6W (b) Higher magnification of white frame in “6W (a)” figure. Fibrous connective tissues on P(LA/CL). Undifferentiated mesenchymal cells, red blood cells and body fluid in pores of HAp. Note: P(LA/CL) and HAp bulk showing clear spaces after demineralization (HE). 6W (c) Higher magnification of blue frame in “6W (a)” figure. New bone and immature tissues with undifferentiated mesenchymal cells and red blood cells in pores. B: bone. 12W (a) Total view at 12 weeks. Fibrous connective tissues around P(LA/CL) showing clear spaces after demineralization (HE). Immature tissues in middle area of HAp block. 12W (b) Bone in interconnected pores.

At 3 weeks after the surgery, loose fibrous connective tissues were seen on the P(LA/CL) membrane, which was recognized as a white bundle of clear space in HE. Capillaries including red blood cells and body fluid were observed in the upper and middle pores of HAp (Fig. 3; 3W). At 6 weeks, P(LA/CL) was clearly seen as a white bundle of clear space. New bone formation was found locally in the pores nearby the outer cortical bone, and the bottom of HAp block was connected directly with the skull (Fig. 3; 6W). The P(LA/CL)/HAp was totally encapsulated by fibrous connective tissues (Fig. 3; 6W (a,b)). In the middle area of HAp, apart from the surface of skull, new bone and immature tissues with undifferentiated mesenchymal cells and red blood cells were found in the pores (Fig. 3; 6W (c)). At 12 weeks, the P(LA/CL)/HAp was encapsulated by fibrous connective tissues (Fig. 3; 12W (a)). Bone formation occurred in the interconnected pores (Fig. 3; 12W (b)). The wound rupture did not occur during 12 weeks.

2. Histological Findings in Collagen /HAp on Skull without Periosteum

**Figure 4:**
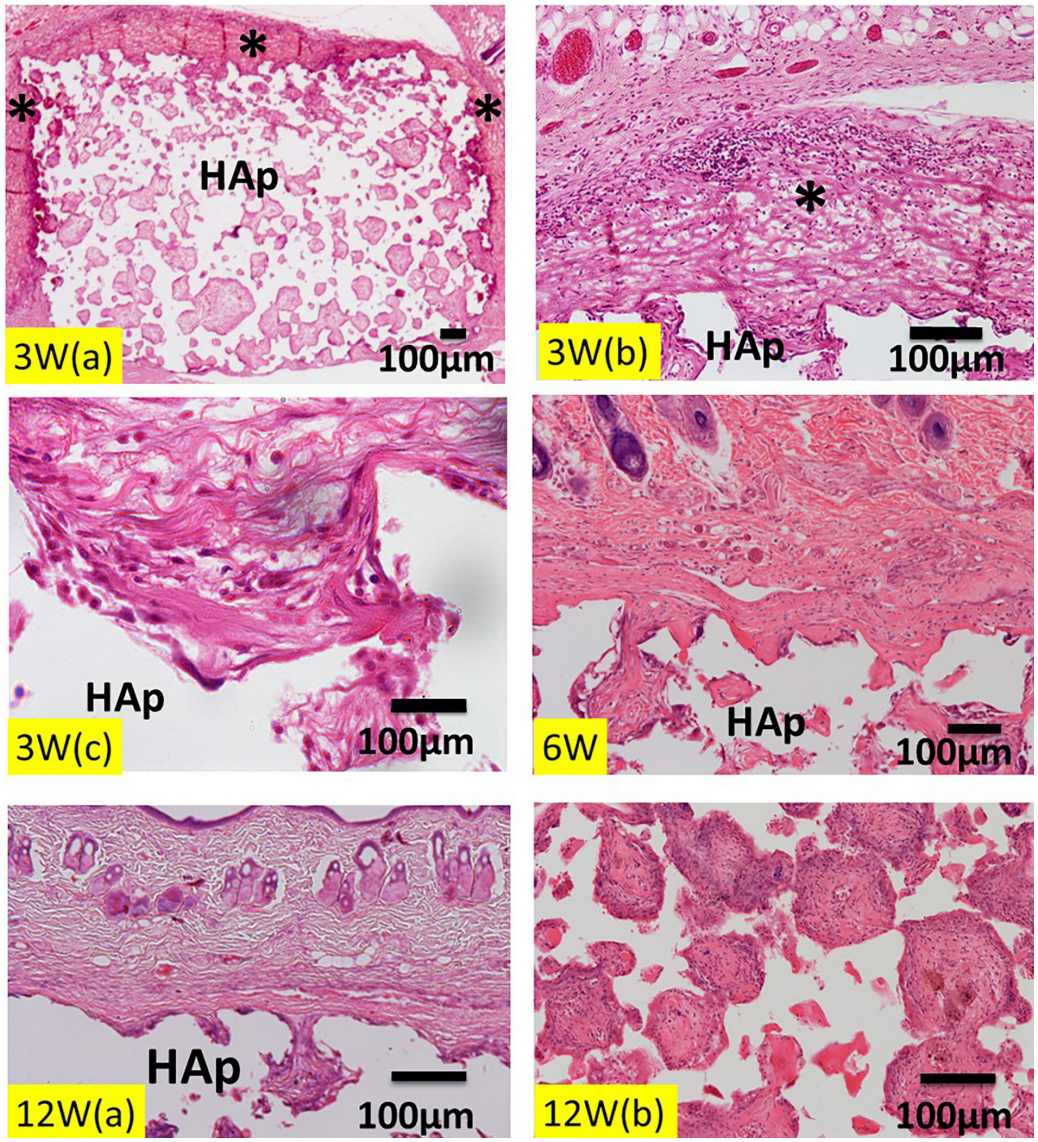
Histological images of collagen / HAp on skull without periosteum. 3W (a) Expansion of collagen membrane (*). Eosin-stained collagen fibers of membrane. Note: Thin thickness of collagen membrane on corners of HAp block. 3W (b) Strongly eosin-stained collagen fibers (*), compared to original connective tissues. Hematoxylin-stained white blood cells in upper layer of membrane. 3W (c) Wavy collagen fibers of membrane in top pore of HAp. *: collagen membrane. 6W; Disappearance of collagen membrane. Fibrous connective tissue formation in upper pores. 12W (a) Disappearance of collagen membrane. HAp covered with fibrous connective tissues. 12W (b) Dense connective tissues in pores of middle area of HAp block.

At 3 weeks after the surgery, the collagen membrane was remarkably expanded except the upper corners of the HAp block (Fig. 4; 3W (a)). The fibers of the collagen membrane were stained strongly by eosin, and the collagen membrane on the corners of HAp was extremely thin in thickness (Fig. 4; 3W (a)). Strongly eosin-stained collagen fibers were distinguished from pinkish fibers of the original connective tissues. Hematoxyline-stained white blood cells infiltrated into the upper layer of the expanded membrane (Fig. 4; 3W (b)). The wavy collagen fibers of the membrane were seen inside the top pores of HAp (Fig. 4; 3W (b,c)). Immature tissues including undifferentiated mesenchymal cells, red blood cells and tissue fluid were found in the middle pores of HAp (Fig. 4; 3W (a)). At 6 weeks, the collagen membrane disappeared, and fibrous connective tissues were formed in the upper pores (Fig. 4; 6W). Strongly eosin-stained collagen fibers were not found. At 12 weeks, residues of the collagen membrane were not seen (Fig. 4; 12W (a)). The HAp block was encapsulated by fibrous connective tissues (Fig. 4; 12W (a)). The wound rupture did not occur during 12 weeks.

3. Histomorphometric Measurement

The upper half area of HAp block was decided as a concerning area for the measurement, because bone conduction occurred in the pores of the bottom area of HAp block on the skull. The average percentage of bone in the P(LA/CL)/HAp increased with time, while bone formation was not found in the upper half area of the collagen/HAp. Bone in the P(LA/CL)/HAp at 6 and 12 weeks occupied 8.3 % and 10.0 %, respectively. The percentage of immature tissues in the P(LA/CL)/HAp revealed significantly higher than that in the collagen/HAp at 3, 6, and 12 weeks. On the other hand, the percentage of connective tissues in the collagen/HAp revealed significantly higher than that in the P(LA/CL)/HAp at 3, 6, and 12 weeks ( Table 1).

## Discussion

The synthetic P(LA/CL) bilayer membrane, an absorbable and polymeric material, had a better performance in the barrier effects against soft tissues than the porcine skin-derived collagen bilayer membrane. The P(LA/CL) membrane remained until 12 weeks later, while the collagen membrane was totally expanded at 3 weeks and almost absorbed until 6 weeks in this onlay model. The *in vivo* appearance of the P(LA/CL) membrane at 12 weeks was compatible to the *in vitro* results indicating 40% degradation rate of the membrane in the dissolution test for 12 weeks in phosphate-buffered saline (5).

1. Animal model

Generally, adult-senior stage patients lose teeth and alveolar bone with periosteum, mainly due to periodontal diseases, and require vertical and/or horizontal bone augmentation for oral rehabilitation. Until now, almost all *in vivo* studies were done in bone defect (inlay) models with periosteum in young-adult rats (10,11). Vertical or horizontal augmentation model is not dynamic more than bone defect (inlay) model consisting of 3-4 bony walls. The supply of mesenchymal stem cells and blood was much better in bone defect models than in augmentation models on cortical bone. In this study, therefore, the application of the barrier membranes was used as vertical bone augmentation (onlay) model without periosteum of 10–12-months old rats (adult-senior stage) for GBR. We believe that the present model is biologically similar and biomimetic to the real adult-senior staged patients requiring bone augmentation for dental implant placements.

2. Barrier Membranes

Native collagen-based materials have been commonly applied as absorbable barrier membranes for GBR (2-4,12-14). Collagen is a main component in human body, and the collagen-based materials have an outstanding biocompatibility (15-18). However, it is difficult to control the absorbability and strength of animal-derived collagen materials. It was reported that the non-cross-linked collagen membrane (Bio-Guide®) consisting of type I and III collagen was digested by collagenase, and disappeared generally during 4-8 weeks (12,13). Interestingly, as a dry collagen material has a characteristic of high potential of water retention (wettability), the wet expansion of the collagen membrane occurred spontaneously in scalp tissues in the present study. The collagen membrane presents a mesh-like structure, hydrophilic and permeable in aqueous environments, allowing greater cell infiltration (5,6). The first biological interaction that occurs in body is the adsorption of plasma proteins at the membrane–tissue interface. Proteins have more affinity to accumulate at the interface of barrier membrane, and surface wettability also affects platelet adhesion/activation, blood coagulation, cell and bacterial adhesion (14). On the other hand, the P(LA/CL) membrane was developed as a prolonged barrier membrane against connective tissues for GBR, composed of both the solid layer inhibiting cellular and bacterial infiltration and the multi-porous layer, and degraded by hydrolysis in about 20 weeks (5,6). Hydrolysis of PCL occurs by end-chain scission because of its highly crystalline chain structure. Hence, hydrolysis of PLA occurs at random points (5). In addition to the slower degradation, another advantage of blending PCL is that it produces a less acidic environment during its degradation (4). The slow degradation prevents the accumulation of by-products in the tissue microenvironment, thereby avoiding an overly acidic pH (19). The P(LA/CL) membrane remained almost intact as a barrier until 12 weeks later, while the collagen membrane was expanded by body fluid such as blood and tissue liquid until 3 weeks. Moreover, neutrophils infiltrated into the upper layer of expanded collagen fibers at 3 weeks. The residues of the collagen membrane were not observed at 6 and 12 weeks. Until 6 weeks, fibroblasts proliferated in the swelled collagen fibers and should produce MMPs, especially collagenase, in the deformed collagen membrane. The enzymatic activity of fibroblasts, neutrophils and macrophages causes the collagen membrane to rapidly digest, and the collagenous material was replaced by newly formed collagen. The changes in the barrier morphology should correlate strongly with the migration and invasion of fibroblasts, necessary for fibrous tissue formation, from gum and mucosa. In contrast, the P(LA/CL) membrane remained clearly until 12 weeks in this augmentation model. It was reported that the weight of the P(LA/CL) membrane decreased in 45% after 26 weeks in the *in vitro* dissolution test, using phosphate-buffered saline at 37°C (4). Moreover, to investigate the degradability of the P(LA/CL) membrane *in vivo*, P(LA/CL) membranes were implanted into rat subcutaneous tissues, and the results showed P(LA/CL) remained clearly until 24 weeks, and maintained an efficient barrier function (20). Our results in the present study agree with the previous reports (5,20). The lower degradation rate of the P(LA/CL) membrane was a better performance in a barrier function than the collagen membrane. In addition, conventional tensile tests showed that the P(LA/CL) membrane had a significantly smaller tensile strength compared with poly (lactic-glycolic acid: PLGA) membrane (GC membrane, GC Corp, Tokyo, Japan) (5). In contrast, the breaking strain of the P(LA/CL) membrane was more than twenty times greater than that of the PLGA membrane, indicating that the P(LA/CL) membrane underwent a greater deformation prior to the rupture. Unfortunately, very prolonged residual P(LA/CL) membrane might cause to wound rupture and chronic inflammation. In near future, the absorption of improved barrier membranes for GBR will be harmonized with bone-forming process in bone augmentation/regeneration.

3. Bone Managements for Bone Formation in Porous Scaffolds

The GBR procedure needs both an adequate scaffold that provides cell-anchorage and sufficient mechanical support and a barrier membrane that inhibits cellular invasion for vertical and/or horizontal augmentation in bone volume loss. It is well known that highly porous scaffolds promote bone formation better than the dense type (21,22). A non-porous solid biomaterial acts like a material’s wall that inhibits both cellular and capillary invasion. In the P(LA/CL)/HAp, bone was locally formed in the middle and lower areas of the HAp block at 6 weeks. The immature tissues were observed in the upper pores of HAp at 12 weeks. Bone formation was not found in the upper pores of HAp even at 12 weeks, although the HAp block was 75% porosity. It was suspected that the main cause was insufficient supplies of mesenchymal stem cells and growth factors from highly calcified cortical bone of aged rats. We believe, therefore, cortical perforations into bone marrows or ultrasonic-scratching technique into the outer cortical plate of skull will contribute to an accelerated bone formation in GBR. The bone managements should contribute to the supply of bone marrow cells and the release of bone matrix- and blood-derived growth factors from the original bone (23-25). The marrow-derived stem cells will proliferate and differentiate into osteoblasts in the interconnected pores of HAp block and in the porous layer of the P(LA/CL) membrane by reacting with several growth factors from bone and blood. Especially, bone morphogenetic proteins (BMPs) in bone matrix are key growth factors for accelerated bone formation in GBR procedure. BMPs has a strong bone-inducing capability, and vertical bone augmentation was achieved at 3 weeks by onlay implant using the composite of recombinant human BMP-2 (10 μg) and atelocollagen material (10 mg) on adult-senior staged rat skull model without periosteum (9).

## Conclusions

The barrier effects of the P(LA/CL) membrane and the collagen membrane were evaluated histomorphometrically in the vertical augmentation model on 10–12-months old rat skull without periosteum. The P(LA/CL) membrane could achieve barrier effects under the repositioned soft tissues until 12 weeks and local bone formation occurred in the interconnected pores of HAp at 6 weeks. On the other hand, the collagen membrane did not inhibit cellular invasion for its expansion at 3 weeks and was absorbed until 6 weeks. The results indicated that the P(LA/CL) membrane might be effective in GBR procedure. Further investigation will be needed for the improvement of absorption rate and structure of the P(LA/CL) bilayer membrane.

## Figures and Tables

**Table 1:** Changes in percentage of each tissue to total volume.

Explant	3 weeks				6 weeks				12 weeks			
Tissue (%)	HAp	B	IT	CT	HAp	B	IT	CT	HAp	B	IT	CT
P (LA/CL)	28.0±2.2	0	65.7±4.2*	6.3±2.6	27.7±2.1*	8.3±2.4*	50.7±4.2*	13.3±4.7	26.3±2.6	10.0±0.8*	45.7±4.1*	18.0±4.2
Collagen	28.3±1.7	0	48.3±1.7	23.3±2.6*	28.3±2.4	0	3.3±4.7	68.3±6.2*	28.0±2.2	0	0	72.0±2.2*

Hap, hydroxyapatite; B, bone; IT, immature tissues; CT, connective tissues. Total volume is designated as 100%. Values: mean±SD; n=3. *significant difference *p*<0.05

## Data Availability

The datasets used and/or analyzed during the current study are available from the corresponding author.
